# Chemical ecology of willows (*Salix* L.): ecological roles, evolutionary dynamics and phytochemical diversity

**DOI:** 10.1093/aob/mcag235

**Published:** 2026-07-24

**Authors:** Martin Volf, Priscila Mezzomo, Sofian A Renoult, Natascha D Wagner, Andrea Calvijo McCormick

**Affiliations:** Biology Centre CAS, Institute of Entomology, Branisovska 31, Ceske Budejovice 37005, Czech Republic; Faculty of Science, University of South Bohemia, Branisovska 31, Ceske Budejovice 37005, Czech Republic; Biology Centre CAS, Institute of Entomology, Branisovska 31, Ceske Budejovice 37005, Czech Republic; Forest Health and Biotic Interactions, Swiss Federal Institute for Forest, Snow and Landscape Research (WSL), Birmensdorf 8903, Switzerland; Biology Centre CAS, Institute of Entomology, Branisovska 31, Ceske Budejovice 37005, Czech Republic; Faculty of Science, University of South Bohemia, Branisovska 31, Ceske Budejovice 37005, Czech Republic; Department of Systematics, Biodiversity and Evolution of Plants (with Herbarium), University of Goettingen, Göttingen 37073, Germany; School of Agriculture and Environment, Massey University, Palmerston North 4442, New Zealand

**Keywords:** Defence, herbivory, flavonoids, mycorrhiza, phenolic glycosides, plant–insect interactions, pollination, salicinoids, tannins, volatile organic compounds

## Abstract

**Background:**

The genus *Salix* comprises ∼450 species that are known for rich and distinctive specialized chemistry, dominated by phenolic compounds, such as salicinoids, flavonoids and tannins, in addition to diverse volatile organic compounds. These metabolites mediate interactions with herbivores, pathogens, mutualists and the abiotic environment. Owing to their species richness, frequent hybridization, polyploidy and diverse ecological interactions, willows represent an exceptional model for studying the evolution and ecological functions of plant specialized metabolites.

**Scope:**

We synthesize current knowledge on the ecological and evolutionary roles of willow specialized metabolites, integrating evidence from chemical ecology, metabolomics, phylogenetics and evolutionary biology. We examine how non-volatile and volatile chemistry shapes willow interactions with antagonists and mutualists and how these interactions vary across environmental gradients. We focus on evolutionary trends in willow chemistry in response to various selection pressures and explore the effects of hybridization, introgression and polyploidization on willow metabolomic profiles. We discuss emerging insights into chemical trait syndromes, metabolic constraints and the role of abiotic filtering versus biotic selection in shaping phytochemical variation in willows.

**Conclusions:**

Willows combine extraordinary chemical diversity with high evolutionary flexibility, which is likely to drive their success across contrasting environments. Variation in their specialized chemistry emerges from the interplay between selection, imposed by herbivores, pathogens and mutualists, and environmental filtering along climatic and resource gradients. These selective forces act on both metabolite concentration and structural variation in willow metabolomes, while hybridization, polyploidy and constraints within metabolic pathways modulate both the evolutionary trajectories and the impact of willow chemistry on their ecological interactions. Advances in untargeted metabolomics and phylogenomics now enable a more holistic understanding of willow chemical strategies beyond a small set of canonical compounds. As such, willows remain a powerful model system for uncovering general principles governing the evolution, function and diversification of plant specialized metabolites.

## INTRODUCTION

The genus *Salix* L. (willows) comprises ∼450 species and is widely distributed, with a particular richness at higher latitudes of the Northern Hemisphere (Fig. 1; Skvortsov, 1999; Argus, 2010). The species are dioecious and include trees, shrubs and dwarf shrubs, with high intra- and interspecific plasticity of phenotypical traits. China is a centre of diversity, with ∼200–250 species; >160 species occur in Eurasia and ∼140 in North America (Skvortsov, 1999; Argus, 2010). Notably, *Salix* exhibits high species diversity even in regions that otherwise support relatively species-poor woody floras. In Europe, for example, >60 species of *Salix* are recognized (Myklestad and Birks, 1993; Skvortsov, 1999). This is approximately three to ten times more than in other species-rich tree-forming genera, such as *Acer*, *Betula*, *Quercus* or *Pinus*, and is exceeded only by the largely shrubby genera *Rubus* and *Rosa* (Warburg, 1964; Kurtto *et al.*, 2004, 2010; Gao *et al.*, 2020).

**Fig. 1.**
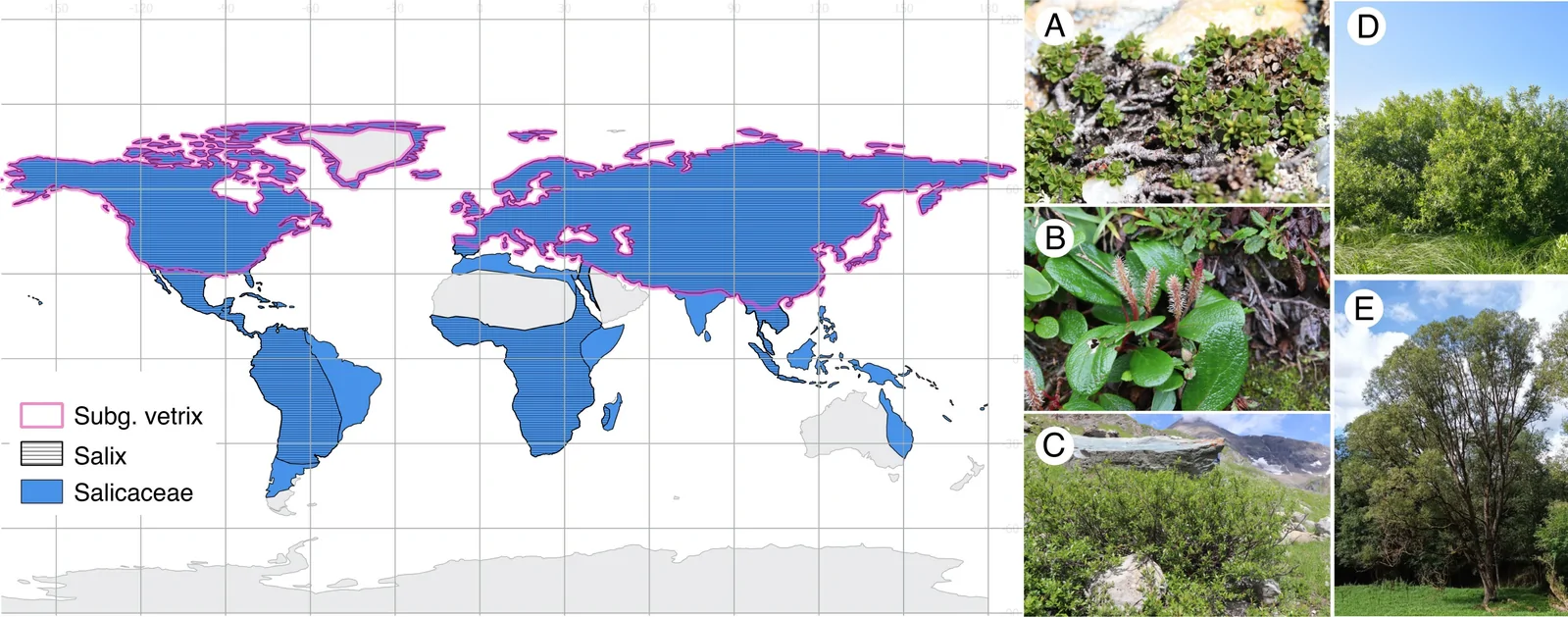
Willows have a broad geographical distribution from tropical to arctic regions. They are particularly diverse in the Northern Hemisphere, which harbours both subg. *Salix* and the diverse *Vetrix* clade. Individual species occupy a broad range of habitats and show various growth forms, including dwarf species, such as *Salix serpillifolia* (A) or *Salix reticulata* (B), small to large shrubs, such as *Salix mielichhoferi* (C) or *Salix eriocephala* (D), and large trees, such as *S. fragilis* (E). The map is based on distribution maps and data reported by Wu *et al.* (2015) and Stevens (2001 onwards).

Willows occupy a broad ecological range, occurring from floodplains of major rivers to alpine and subarctic environments, where they often constitute dominant structural and functional components of vegetation (Hörandl *et al.*, 2012; Wagner *et al.*, 2021). Owing to their abundance and species richness, many *Salix* species function as keystone taxa within plant communities, sustaining diverse assemblages of associated organisms, especially insects (Price, 2005; Narango *et al.*, 2020). Furthermore, willow leaf litter contributes substantially to nutrient cycling in both terrestrial and aquatic ecosystems (González *et al.*, 2022). These ecological interactions and processes are profoundly influenced by the array of specialized (secondary) metabolites produced by *Salix*, which mediate biotic relationships and underpin many adaptive and ecological characteristics of the genus (Šlapokas and Granhall, 1991; Pasteels and Rowell-Rahier, 1992; Boeckler *et al.*, 2011).

Owing to their distinctive and bioactive specialized metabolites, willows have attracted human attention for >3500 years. Extracts from willow bark have long been used in traditional medicine to alleviate pain and inflammation (Montinari *et al.*, 2019). The first formal experimental report on fever-reducing properties of willow bark was carried out by Edward Stone in 1763, followed by isolation and identification of salicin and salicylic acid, finally leading to the synthesis of acetylsalicylic acid and the commercial production of aspirin in 1899 (Montinari *et al.*, 2019). Experimental evidence for ecological roles of specialized metabolites in *Salix* emerged much later (e.g. Rowell-Rahier, 1984*b*; Tahvanainen *et al.*, 1985). Since then, willows have been recognized as one of the key models in chemical ecology that uniquely combines rich biotic interactions, broad ecological valence and diverse evolutionary patterns, including frequent polyploidization and hybridization (e.g. Pasteels and Rowell-Rahier, 1992; Volf *et al.*, 2023; Leong *et al.*, 2024; Marinček *et al.*, 2025; Renoult *et al.*, 2025). In this review, we aim to integrate the different aspects of willow ecology and evolution to illustrate how they both shape and are shaped by the specialized chemistry of willows.

## NON-VOLATILE METABOLITES AND THEIR ROLES IN PROTECTING WILLOWS AGAINST BIOTIC AND ABIOTIC STRESS

Owing to their broad geographical distribution, willows possess rich chemistry that helps them cope with diverse abiotic and biotic conditions in the environments they inhabit (Volf *et al.*, 2023; Leong *et al.*, 2024). Willow specialized chemistry is based primarily on various phenolics generated by the phenylpropanoid pathway (Fig. 2). Phenolics found in willows include a broad diversity of flavonoids, tannins, phenolic acids, other simple phenolics and phenolic glycosides, such as salicinoids, which, together, can comprise ≤20 % of dry leaf mass (Julkunen-Tiitto, 1989; Boeckler *et al.*, 2011; Nissinen *et al.*, 2018; Volf *et al.*, 2023). Apart from phenolics, willows also produce non-phenolic glycosides, sterols and terpenes (Tawfeek *et al.*, 2021). Out of these metabolites, flavonoids, condensed tannins (proanthocyanidins) and salicinoids have received most of the attention owing to their bioactivity and the physiological and ecological roles they play in willows, including their contribution to willow resistance to both abiotic and biotic stress (Fig. 2). As dioecious plants, willows can show significant sex-specific differences in their investment in different phenolic compounds. These differences have been documented best in flavonoids and salicinoids (Sivadasan *et al.*, 2015; Nissinen *et al.*, 2016; Keefover-Ring *et al.*, 2022; Ramstack Hobbs *et al.*, 2022), whereas sexual dimorphism in condensed tannins seems to be less prominent based on current evidence (Yang *et al.*, 2020; Ramstack Hobbs *et al.*, 2022).

**Fig. 2.**
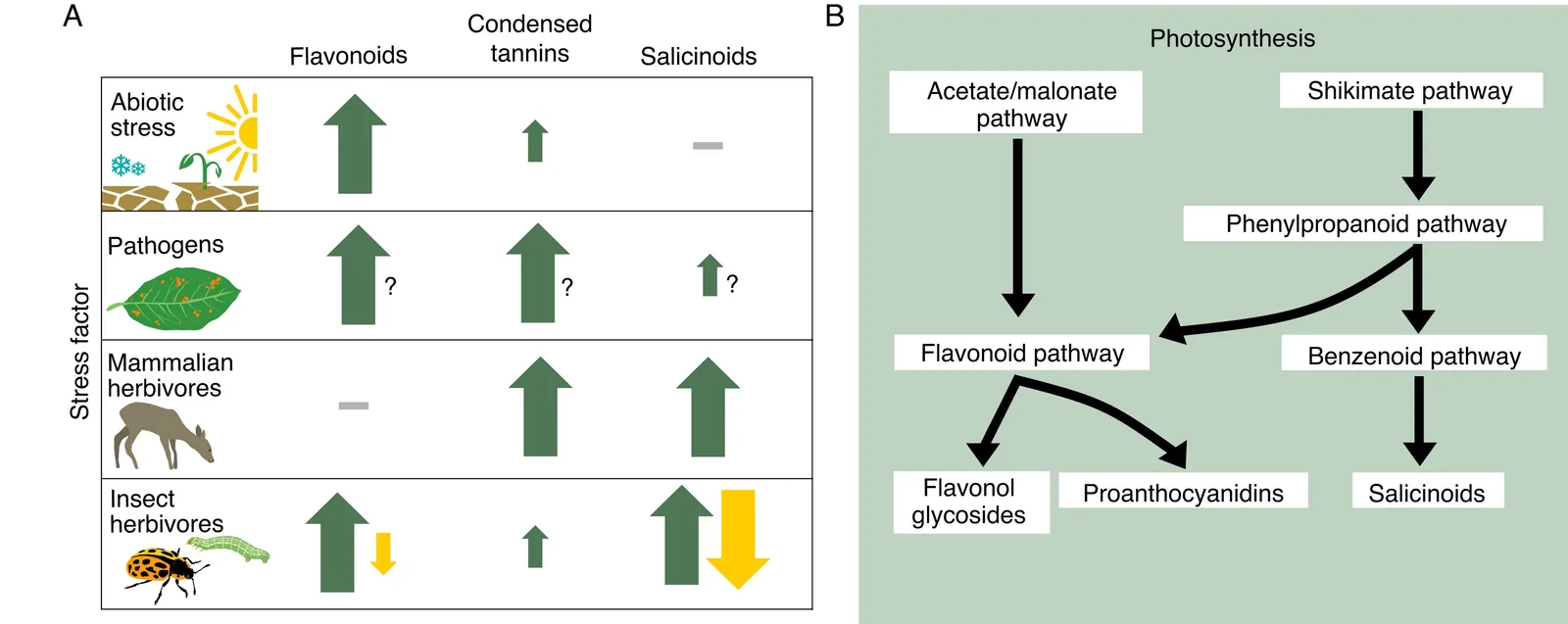
Conceptual summary of the roles of flavonoids, condensed tannins (proanthocyanidins) and salicinoids in willow resistance to abiotic stress and natural enemies (A) and a simplified diagram showing metabolic pathways producing these metabolites (B). In A, green upward-pointing arrows indicate positive effects of individual metabolites on willow performance under the respective stressors. Yellow downward-pointing arrows indicate negative effects, which may arise from antimicrobial properties that benefit insect herbivores or from the ability of specialized insect herbivores to sequester these compounds. Arrow size reflects the relative magnitude of each relationship as suggested by the available literature. Question marks indicate relationships for which evidence from willows remains limited. Grey lines indicate interactions that do not appear to play a major role in willows based on current knowledge.

The flavonoids found in willows show wide structural diversity and include flavones, flavonols, flavanones, dihydroflavonols, isoflavones, flavan-3-ols and anthocyanins (Tawfeek *et al.*, 2021). Flavonoids are found in various willow tissues, with their concentrations typically being highest in leaves and buds, while also being present in bark, catkins, stems or roots (Tawfeek *et al.*, 2021; Keefover-Ring *et al.*, 2022; Curtasu and Nørskov, 2024; Mezzomo *et al.*, 2025). Flavonoids perform diverse functions in plant tissues, acting as antioxidants, contributing to protection against drought and ultraviolet (UV)-induced stress, and providing defence against insect herbivores (Treutter, 2006). In willows, flavones and flavonols, including luteolin glycosides, myricetin derivatives and hyperin, have been particularly associated with responses to UV radiation (Tegelberg and Julkunen-Tiitto, 2001; Sivadasan *et al.*, 2015). The responses seem to be more pronounced in females, suggesting that they might invest relatively more into their protection against UV stress than males (Sivadasan *et al.*, 2015). This interpretation is further supported by the higher flavonoid content reported in female willow leaves and catkins, where these metabolites might serve protective roles against both UV stress and pathogens (Nissinen *et al.*, 2016; Keefover-Ring *et al.*, 2022). In comparison to UV-induced responses, responses in flavonoid content induced by water stress are typically more variable and species specific, including both up- and downregulation of various flavones, flavanonols and flavanones and their glycosides (Köhler *et al.*, 2020).

In interactions with insect herbivores, flavonoids can deter feeding or oviposition, retard larval growth, hamper food conversion and interfere with immunity or hormone signalling (Oberdörster *et al.*, 2001; Simmonds, 2001; Onyilagha *et al.*, 2004; Shi *et al.*, 2020; Bai *et al.*, 2024). In willows, luteolin derivatives contribute to resistance against the specialist leaf beetle *Phratora vulgatissima*, particularly in synergy with salicinoids (Torp *et al.*, 2013). In contrast, myricetin seems to be positively correlated with the growth of generalist *Operophtera brumata* caterpillars (Ruuhola *et al.*, 2001). Potentially, this could link to anti-microbial effects of flavonoids that have been shown to improve caterpillar performance in other plant systems (Zhang *et al.*, 2019). Effects of willow flavonoids on herbivore gut microbiota remain untested. However, several compounds isolated from willows show antimicrobial activity in laboratory assays (González-Alamilla *et al.*, 2019; Salih *et al.*, 2024), while also probably protecting willows from fungal pathogens (Hakulinen *et al.*, 1999; Keefover-Ring *et al.*, 2022).

Willows are also rich in tannins, particularly proanthocyanidins (condensed tannins) (Topp *et al.*, 2002; Volf *et al.*, 2022). Proanthocyanidins are oligomers or polymers of flavonoids and share some of their ecological functions in willows. They serve as antioxidants and are likely to protect willows from UV radiation (Randriamanana *et al.*, 2015; Ramos *et al.*, 2019), although UV-induced increases in proanthocyanidins are typically weaker than those observed for flavonoids, suggesting a more limited role in this respect (Tegelberg and Julkunen-Tiitto, 2001; Tegelberg *et al.*, 2003). Like flavonoids, willow proanthocyanidins exhibit strong antimicrobial activity (Salih *et al.*, 2024). Elevational clines in proanthocyanidin concentrations have been shown to parallel elevational patterns in damage by *Melampsora* rust (Volf *et al.*, 2022). Their antimicrobial properties are also likely to contribute to the slow decomposition of leaf litter from some willow species, particularly during the initial weeks after leaf fall (Šlapokas and Granhall, 1991; Hoeber *et al.*, 2020). Subsequently, tannins are leached to the soil, where they may affect metal ion or nitrogen availability to microbes or other decomposers, with the speed of leaching being negatively correlated with their molecular weight (Schofield *et al.*, 1998).

Proanthocyanidins also show strong capacity for precipitating proteins (Salminen and Karonen, 2011). This capacity occurs primarily in acidic gut conditions and can reduce protein digestibility in mammalian herbivores feeding on willows (Spaeth *et al.*, 2002; Heiska *et al.*, 2007). In response, some large herbivores, such as moose and beaver, have evolved salivary proteins that bind specifically to the linear proanthocyanidins common in willows (Hagerman and Robbins, 1993). Proanthocyanidins also defend willow seedlings from slugs (Orians *et al.*, 2013). Their deterrent effects on insects, however, appear less consistent and can be weaker than those of other willow specialized metabolites (Kelly and Curry, 1991; Ayres *et al.*, 1997; Volf *et al.*, 2015*a*; Leong *et al.*, 2024). This might be attributable to relatively high pH in the guts of some insects, such as lepidopteran caterpillars, which limits the protein precipitation capacity of proanthocyanidins (Salminen and Karonen, 2011). Instead, caterpillars often respond more strongly to oxidatively highly active hydrolysable tannins (Segar *et al.*, 2017), which, however, are only rarely found in willows (Volf *et al.*, 2022).

Salicinoids comprise a relatively small group of ∼35 salicyl alcohol-containing phenolic glycosides that have attracted considerable scientific interest because of their notable bioactivity and their important roles in willow ecology (Julkunen-Tiitto, 1989; Boeckler *et al.*, 2011; Keefover-Ring *et al.*, 2014). Their biosynthesis is thought to proceed through benzenoid intermediates, including salicyl benzoate and related glucosides (Fellenberg *et al.*, 2020; Gordon *et al.*, 2022). Salicinoids are largely characteristic of willows and their sister genus *Populus* (poplars), both of which belong to the subfamily Salicoideae (Palo, 1984; Keefover-Ring *et al.*, 2014). Tropical members of this subfamily, such as *Idesia* and *Itoa*, primarily produce phenolic glycosides that are structurally similar to salicinoids, such as itosides, or close salicinoid analogues (Chai *et al.*, 2008; Feistel *et al.*, 2017; Nie *et al.*, 2025). Interestingly, salicin has also been reported from *Homalium*, a basal genus within Salicoideae, suggesting that salicinoid biosynthesis is ancestral to the subfamily (Liu *et al.*, 2013; Ogutcen *et al.*, 2024).

In general, salicinoids accumulate to high levels in young, developing tissues, although their seasonal dynamics are highly variable (Julkunen-Tiitto, 1989; Boeckler *et al.*, 2011; Mezzomo *et al.*, 2025). They occur predominantly in leaves, flowers and bark (Palo, 1984; Julkunen-Tiitto, 1989; Boeckler *et al.*, 2011) and seem to reach higher concentrations in male than in female willows (Nissinen *et al.*, 2016; Keefover-Ring *et al.*, 2022). In poplars, they are also present in roots (Hillabrand *et al.*, 2024). In willows, the occurrence and the ecological and physiological functions of salicinoids in below-ground tissues remain poorly characterized. Salicinoid levels change in response to abiotic stress (Turtola *et al.*, 2005), but they do not seem to play a pronounced direct role in protecting willows against adverse abiotic conditions (Tegelberg and Julkunen-Tiitto, 2001; Tegelberg *et al.*, 2003).

Salicinoid-rich willow extracts exhibit antibacterial and antiviral activity *in vitro*, often probably in synergy with other phenolics (Tienaho *et al.*, 2021; Salih *et al.*, 2024). Phenolic glycosides, including salicinoids, have also been reported to be negatively correlated with rust infection in *Salix triandra* (Hjältén *et al.*, 2007). Hydrolysis of salicinoids releases salicyl alcohol and may, in principle, feed into salicylic acid-related metabolism (Julkunen-Tiitto and Meier, 1992). However, although salicylic acid displays direct antifungal activity and serves as a central defence hormone (Vlot *et al.*, 2009), a defined catabolic pathway linking salicinoids to salicylic acid-mediated immunity in willows has not been demonstrated. Consequently, the roles of salicinoids in willow–pathogen interactions remain incompletely resolved, particularly *in planta*. Results from poplars suggest that their role is likely to be less pronounced than the roles of flavonoids or proanthocyanidins (Ullah *et al.*, 2017).

Many mammalian herbivores, such as hares, voles or elks, that feed on willows and poplars during winter are deterred by salicinoids (Bryant and Kuropat, 1980; Tahvanainen *et al.*, 1985; Bailey *et al.*, 2007; Heiska *et al.*, 2007). Salicinoids contained in willow bark or twigs reach the highest concentration during winter, which might underlie their roles as anti-herbivore defences during this period (Förster *et al.*, 2010). These herbivores are not only deterred by the bitter taste of salicinoids, but they also respond to volatiles originating from salicinoids, such as 6-hydroxycyclohex-2-en-1-one (Reichardt *et al.*, 1990; Clausen *et al.*, 2010). In contrast, beavers seem to have adapted to salicinoids, and their food choice is unaffected by salicinoid content (Basey *et al.*, 1990; Bailey *et al.*, 2004). Studies from willows that would identify the exact mechanisms by which willow salicinoids affect mammalian herbivores after digestion are rare, and their effects are generally difficult to separate from those of other phenolics contained in the leaves. Possible mechanisms include enzymatic hydrolysis to salicyl alcohol and the formation of reactive breakdown products, but direct *in vivo* demonstrations remain limited (Reichardt *et al.*, 1990; Julkunen-Tiitto and Meier, 1992; Martinsen *et al.*, 1998).

The primary role of salicinoids in willows appears to be defence against herbivorous invertebrates, predominantly insects but also slugs, which can be important herbivores of willow seedlings (Fritz *et al.*, 2001; Ikonen, 2002; Boeckler *et al.*, 2011). This is also supported by results from *Idesia*, a more basal member of the Salicoideae subfamily, in which salicinoid analogues have been shown to provide defence against caterpillars (Feistel *et al.*, 2017). Some evidence suggests that more complex salicinoids, such as salicortin and tremulacin, can have stronger effects on insect herbivores than salicin, at least at the concentrations at which these compounds typically occur in leaves (Lindroth and Peterson, 1988).

Upon ingestion by insect herbivores, complex salicinoids, such as salicortin and tremulacin, can be degraded through ester cleavage, yielding salicin and the labile 1-hydroxy-6-oxo-2-cyclohexenecarboxylic acid (HCC) moiety, which can be converted further into 6-hydroxycyclohex-2-en-1-one and catechol (Ruuhola *et al.*, 2003; Boeckler *et al.*, 2016). 6-Hydroxycyclohex-2-en-1-one and catechol act as feeding deterrents, and catechol can be oxidized to reactive quinones that reduce leaf nutritive value and might interfere with digestive processes (Ruuhola *et al.*, 2003; Boeckler *et al.*, 2016). In generalist herbivore *Lymantria dispar*, salicin and catechol significantly reduced larval growth, and their combination with benzoic acid also reduced survival, indicating that salicinoid breakdown products can have additive or synergistic negative effects on generalist herbivores (Boeckler *et al.*, 2016). In turn, salicinoids deter various generalist insect herbivores from feeding, retard their larval growth or increase their mortality either directly or indirectly, by prolonging their growth and increasing their exposure to natural enemies (Tahvanainen *et al.*, 1985; Matsuki and Maclean, 1994; Ruuhola *et al.*, 2001; Boeckler *et al.*, 2011). At the community level, high salicinoid concentrations can be associated with lower insect herbivore richness, consistent with the exclusion of non-adapted species from assemblages on willows with high salicinoid content (Volf *et al.*, 2015*b*). Laboratory assays have also shown negative effects of salicinoids on the Salicaceae-specialist willow beetle *Phratora vulgatissima*, although responses of specialist herbivores are variable and often involve tolerance or detoxification mechanisms (Kelly and Curry, 1991; Boeckler *et al.*, 2011; Feistel *et al.*, 2017; Schnurrer and Paetz, 2023). Field observations of negative effects on specialists are rarer, suggesting that in natural conditions, negative effects of salicinoids might be outweighed by other factors that benefit specialists when feeding on willows high in salicinoids (Kearsley and Whitham, 1992; Rank *et al.*, 1998).

In contrast to generalists, some specialized herbivores respond positively to salicinoids (Soetens *et al.*, 1991). Phagostimulant effects of salicinoids have been reported in leaf-beetles from the genera *Chrysomela* or *Phratora* (Rowell-Rahier, 1984*a*; Rank, 1992; Orians *et al.*, 1997). Salicin, salicortin, 2′-*O*-acetyl-salicortin or tremulacin can also stimulate oviposition in leaf-chewing and galling sawflies upon contact with the leaf surface (Kolehmainen *et al.*, 1994; Roininen *et al.*, 1999; Fernández *et al.*, 2019). Galling sawflies radiated on willows and are often highly specialized on individual willow species (Price, 2005; Nyman *et al.*, 2006). Beyond positively responding to salicinoids, they can also manipulate host metabolism, including the production of both salicinoids and flavonoids (Nyman and Julkunen-Tiitto, 2000). Overall, the host choice of gallers associated with willows can show both positive and negative correlations with defensive chemistry of willows, but it is modified by other factors, such as pressure by natural enemies, nutrient content, phenology, or host susceptibility to gall induction (Yamazaki and Ohsaki, 2006; Yamaguchi *et al.*, 2012; Volf *et al.*, 2017; de O Mota *et al.*, 2026).

Some of the specialized insect herbivores have developed adaptations that allow them to sequester salicinoids and use them for their own benefit. These herbivores can cleave salicin and oxidize the produced salicyl alcohol to salicylaldehyde, which they use as a defence against invertebrate predators (Pasteels *et al.*, 1983; Kearsley and Whitham, 1992; Michalski *et al.*, 2008). Sequestration of salicinoids has been recorded from various species of *Chrysomela* and *Phratora vitellinae* leaf-beetles or from *Limenitis archippus* butterflies (Matsuda and Sugawara, 1980; Rank, 1992; Hilker and Schulz, 1994; Prudic *et al.*, 2007; Michalski *et al.*, 2008). Females of *Chrysomela* and *Phratora vitellinae* even incorporate salicin into their eggs, which the neonate larvae sequester (Pasteels *et al.*, 1986). In contrast, other *Phratora* species feeding on willows or poplars rely on autogenous secretions rather than salicylaldehyde, and host shifts outside Salicaceae have been accompanied by loss of salicyl-alcohol oxidase activity in *Chrysomela lapponica*, indicating evolutionary flexibility in leaf-beetle defence strategies (Termonia *et al.*, 2001; Kirsch *et al.*, 2011). Beyond defence, specialists may also benefit nutritionally from the glucose released during salicinoid hydrolysis, which can offset metabolic costs and help sustain their rapid growth on salicinoid-rich willows (Pasteels *et al.*, 1986; Kearsley and Whitham, 1992).

## ECO-EVOLUTIONARY TRENDS IN NON-VOLATILE METABOLITES

Pressure from specialized herbivores is likely to be one of the key factors that have shaped willow chemistry over the course of their evolution (Volf *et al.*, 2015*b*, 2023; Leong *et al.*, 2024). Most insect herbivores feed on closely related host plants, and species-rich plant lineages often support a diverse and specialized herbivore fauna (Forister *et al.*, 2015; Segar *et al.*, 2024). Willows, in particular, have supported radiations of insect herbivores, such as nematine sawflies, and generally harbour high insect herbivore diversity (Price, 2005; Nyman *et al.*, 2006; Narango *et al.*, 2020). Many willow-associated herbivores feed predominantly on Salicaceae but can exploit multiple willow species as hosts (Topp *et al.*, 2002; Leong *et al.*, 2022). Because willows frequently occur as assemblages of several species growing in sympatry, selection by specialized herbivores might have driven chemical divergence and lability within the genus (Volf *et al.*, 2023; Leong *et al.*, 2024). In this respect, willows resemble other species-rich plant genera, such as *Bursera*, *Ficus*, *Inga* or *Piper*, in which chemical differentiation appears to help maintain diverse local assemblages of sympatric congeneric species by reducing the overlap in their herbivore communities and thereby lowering the overall intensity of herbivory (Becerra, 2007; Endara *et al.*, 2017; Volf *et al.*, 2018; Massad *et al.*, 2022). Indeed, in willows the chemical similarity between species is positively correlated with the amount of herbivores they share, which might have supported evolutionary divergence in their chemistry, particularly among species from lowland habitats (Volf *et al.*, 2015*a*; Leong *et al.*, 2022).

The evolutionary flexibility of lowland willow species seems to extend beyond individual traits and classes of metabolites, because frequent evolutionary transitions have also been suggested for whole trait syndromes in the genus (Leong *et al.*, 2024). These syndromes are mainly defined by investment into different types of phenolics, including negative correlations between investment into the richness and concentration of salicinoids and flavonoids, on the one hand, and the concentration and activity of proanthocyanidins, on the other (Fig. 3; Topp *et al.*, 2002; Leong *et al.*, 2024). The effects of syndromes on insect herbivore communities seem to be weaker than the effects of individual traits, indicating that factors other than a role in anti-herbivore defence contribute to their maintenance (Leong *et al.*, 2024). Beyond the possible roles of other factors, such as pathogens or protection against abiotic stress, in the formation of the syndromes, they may also arise from intrinsic constraints within the phenylpropanoid pathway (Fig. 2). These constraints can involve metabolic competition for shared precursor and regulatory crosstalk, whereby activation of proanthocyanidin-specific transcription factors downregulates alternative phenylpropanoid branches, such as salicinoid biosynthesis (Boeckler *et al.*, 2014; James *et al.*, 2017). This is consistent with the view that the remarkable chemical flexibility underlying the evolutionary success of species-rich plant genera, such as willows, is often mediated by regulatory shifts in gene expression controlling existing pathways rather than through major evolutionary innovations (Coley, 2024).

**Fig. 3.**
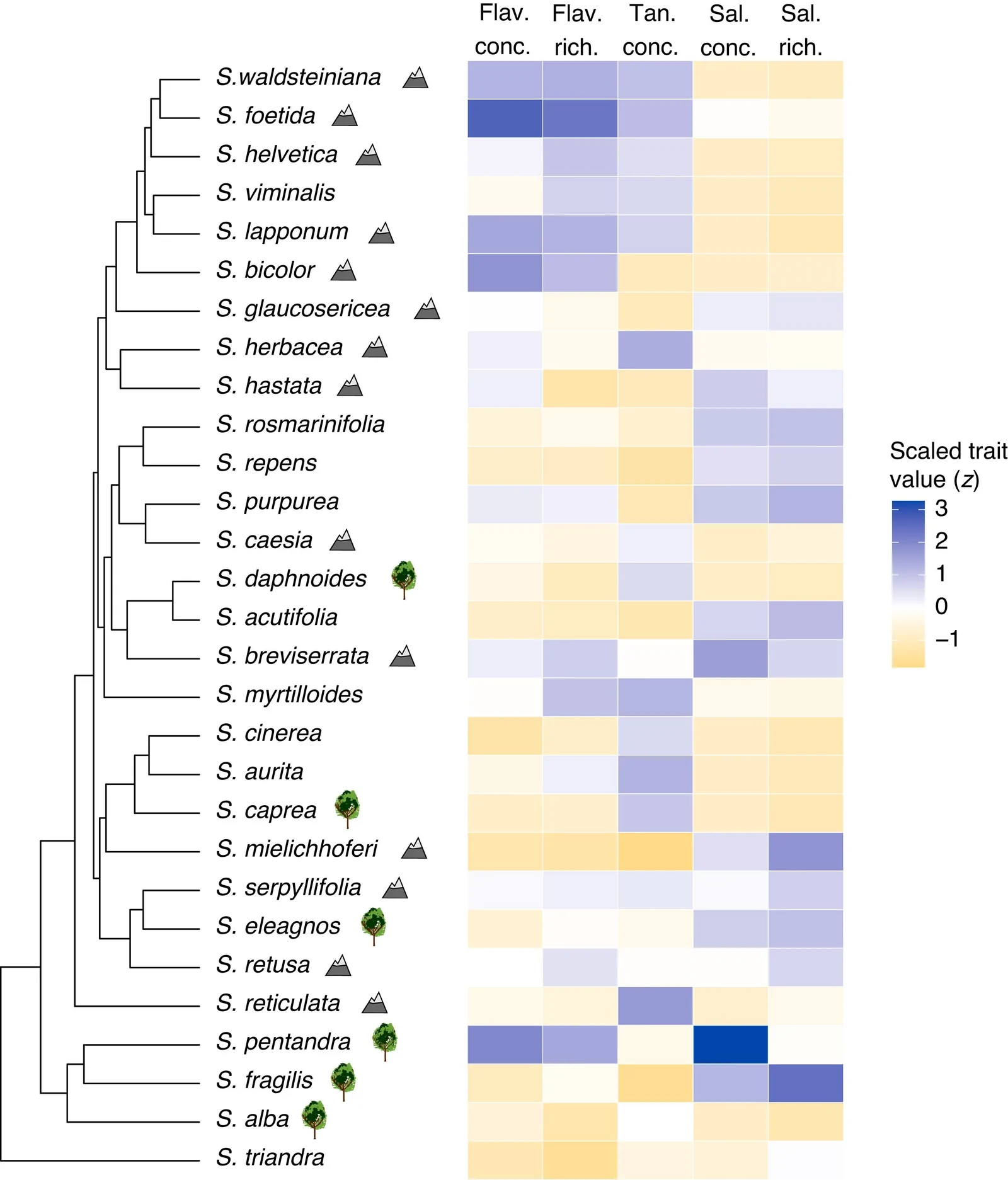
Heatmap of scaled values for flavonoid (Flav.) concentration and richness, condensed tannin (Tan.) concentration and salicinoid (Sal.) concentration and richness mapped onto the phylogeny of 29 Central European willow species (based on data from Volf *et al.*, 2023). The heatmap highlights positive and negative correlations among metabolite groups and shows that several traits exhibit only weak associations with willow phylogeny. Willow morphology and ecology are, like their chemistry, variable, in that willows show various growth forms and elevational distributions, for example. Tree symbols indicate species typically forming trees following Hörandl *et al.* (2012). Other species, such as *Salix triandra* or *Salix viminalis*, may also develop tree forms, but commonly occur as shrubs. Mountain symbols denote species restricted to subalpine and alpine zones in Central Europe and absent from the colline zone and lower elevations following Wagner *et al.* (2021).

In contrast, environments where abiotic stress plays a stronger role appear to filter willows towards reduced chemical variation between species, particularly in metabolites that enhance stress tolerance (Volf *et al.*, 2023). For instance, high-elevation willows can produce high concentrations of flavonoids, but with less structural variation than those found in their lowland relatives. At the interspecific level, this trend is reflected in a phylogenetic increase in flavonoid concentrations among derived highland willow species, accompanied by comparatively limited divergence in their chemical profiles relative to lowland species (Volf *et al.*, 2023). Broader phylogenetic studies could test whether such patterns reflect chemical convergence among high-elevation willow species from distinct clades within the genus. Interestingly, at the intraspecific level, chemical variation sometimes increases with elevation in certain willow species (Volf *et al.*, 2022). Insights from other plant systems suggest that this might result from the high heterogeneity of microhabitat conditions above the treeline or from metabolic disruptions that introduce stochastic variation into the metabolome (Davies *et al.*, 2004; Pellissier *et al.*, 2014; Massatti and Knowles, 2016).

## EFFECTS OF HYBRIDIZATION AND POLYPLOIDY ON NON-VOLATILE METABOLITES

Hybridization is common in *Salix* and has even been reported between species of different taxonomic sections (Skvortsov, 1999; Argus, 2010; Hörandl *et al.*, 2012; Gramlich *et al.*, 2018). The resulting morphological intermediate forms and observed hybrid swarms increase taxonomic difficulties and complicate species delimitation. Nevertheless, hybrid formation is not completely random and was overestimated in previous studies (Hörandl *et al.*, 2012). Backcrossing and introgression are more frequent than hybrid speciation (Gramlich *et al.*, 2018; Marinček *et al.*, 2023; Pittet *et al.*, 2025). Recent studies also found evidence for multiple ancient hybridization events between distinct lineages within the genus (Sanderson *et al.*, 2023; Gambhir *et al.*, 2025).

Owing to their frequent hybridization, willows have been one of the key model systems for studying how introgression influences plant chemistry, performance and biotic interactions (Fritz *et al.*, 1994; Orians, 2000). Similar to other plant taxa, hybridization in willows typically leads to chemical profiles being intermediate between the parents or being similar to one of them (Orians and Fritz, 1995; Orians *et al.*, 2000). The exact inheritance patterns seem to differ between metabolites originating from different metabolic pathways that are regulated by different genes. For example, condensed tannins and salicinoids that originate from different branches of the phenylpropanoid pathway show different inheritance patterns in *Salix eriocephala × sericea* F1 hybrids. These hybrids contain intermediate concentrations of condensed tannins, suggesting predominantly additive inheritance, while the concentration of salicinoids is lower than the parental midpoint, indicating incomplete dominance (Orians *et al.*, 2000).

Beyond their biochemical inheritance, intermediate chemical profiles can have important ecological consequences. In Central Europe, *Salix alba × fragilis* is among the most widespread and abundant willow hybrids, in some regions occurring more frequently than either parental species (Vašut *et al.*, 2024). This hybrid appears to combine the rapid growth and efficient nutrient utilization characteristic of *S. alba* with moderate concentrations of salicinoids inherited from *S. fragilis* (Oberprieler *et al.*, 2013; Ran *et al.*, 2021; Renoult *et al.*, 2025). Such a trait combination might facilitate an optimal balance between growth and defence, because intermediate salicinoid levels are likely to provide sufficient protection against generalist insect herbivores while avoiding the high metabolic costs associated with elevated defensive investment that could otherwise constrain growth (Orians *et al.*, 1997; Kehl *et al.*, 2008; Renoult *et al.*, 2025). Previous studies in other willow systems suggest that such trade-offs are largely transient and occur primarily during the seedling stage, which might represent a critical window for linking hybrid chemical profiles to their abundance in natural populations (Orians *et al.*, 2010).

Hybridization does not only reshape existing metabolite profiles but may also lead to the emergence of entirely novel compounds in willows. Most documented cases of such novel compounds involve flavonoids, which have frequently been the focus of targeted analyses (Förster *et al.*, 2021). The hybrid-specific metabolites range from direct derivatives of parental compounds, through metabolites sharing the same biosynthetic origins without being direct derivatives, to rare modifications, such as sulphated flavanones that are only infrequently recorded in plants (Volf *et al.*, 2015*b*; Noleto-Dias *et al.*, 2020; Förster *et al.*, 2021). Comparative reviews across various plant taxa have estimated that ∼70 % of specialized metabolites are shared between parents and hybrids, ∼25 % occur only in the parents, and ∼5 % are unique to hybrids (Rieseberg *et al.*, 1993; Cheng *et al.*, 2011). A recent untargeted metabolomic study on *Salix alba × fragilis* reported a somewhat higher proportion of hybrid-unique metabolites (∼9 %; Renoult *et al.*, 2025). Consistent with earlier targeted studies, Renoult *et al.* (2025) found that most of these compounds were structurally related to parental metabolites. This structural relatedness spanned the metabolome and multiple compound classes, suggesting that novel metabolites in hybrids might arise primarily through recombination of parental substrates and enzymes, rather than through large-scale reorganization of metabolic pathways (Cheng *et al.*, 2011).

On an evolutionary scale, ancient hybridization and reticulate evolution might have contributed to the distinct chemical profiles observed in some willow species. *Salix reticulata* and *S. daphnoides* are notable examples, both showing particularly high levels of admixture. The former is likely to have originated from a hybridization between circumpolar dwarf shrubs and a more ancestral lineage, whereas the latter belongs to a clade that appears to be of hybrid origin as a whole (Wagner *et al.*, 2023; Marinček *et al.*, 2024). Interestingly, both species were reconstructed as relatively distinct in terms of structural variation in their metabolomes and, in the case of *S. daphnoides*, particularly distinct in structural variation in salicinoids (Volf *et al.*, 2023). Whether these distinctive metabolomic traits reflect their hybrid origin and introgressed chemical profiles remains unresolved. Addressing this question will require further comparative analyses across additional willow species exhibiting similar degrees of admixture.

About 40 % of willow species are polyploid, most of which are assumed to be the result of allopolyploidization and therefore comprise the combination of two distinct parental genomes (Suda and Argus, 1968; Wagner *et al.*, 2020). Interestingly, the whole Salicoid lineage is the result of an ancient genome duplication (Wang *et al.*, 2025). Polyploidy is an important factor in the evolution of most plant lineages (Soltis *et al.*, 2015). Ecological and physiological novelties created by unique gene combinations might have facilitated the adaptation of polyploids to changing environments (Van de Peer *et al.*, 2021), and polyploid species often exhibit greater genetic diversity. This diversity might contribute to their enhanced tolerance of biotic and abiotic stress factors and facilitate survival in harsh conditions and their tendency to occur in colder climates (Parisod and Broennimann, 2016; Rice *et al.*, 2019; Van de Peer *et al.*, 2021).

Willows, with their highly variable ploidy, thus provide an excellent system for investigating how polyploidization shapes plant metabolomes and exploring whether the resulting changes affect plant resistance to climatic stress. Polyploidization can profoundly alter plant chemistry, yet its effects appear to be genotype and lineage specific, making broad generalizations across taxa difficult (Gaynor *et al.*, 2020; Wu *et al.*, 2024). In poplars, polyploidization has been shown to modify metabolomes in ways that are correlated with growth performance (Cheng *et al.*, 2019), probably through altered regulation of genes controlling lignin synthesis and cell expansion, resulting in lower lignin content and larger cells (Xu *et al.*, 2022). Comparable patterns have been observed in willows, where higher ploidy levels are associated with reduced lignin investment and enhanced growth, potentially improving performance of such genotypes in resource-rich conditions (Serapiglia *et al.*, 2015). However, the broader consequences of polyploidization for willow metabolomes, including its impact on the diversity and novelty of specialized metabolites, remain largely unexplored. Given the high variation of ploidy in naturally occurring willow species, including both auto- and allopolyploids, this represents a particularly promising direction for future research. Overall, integrating chemical profiling with ploidy variation and signatures of ancient hybridization, including the formation of allopolyploids, might provide key insights into the processes underlying the extraordinary chemical diversity observed among willow species.

## VOLATILE ORGANIC COMPOUNDS

In addition to non-volatile specialized metabolites, willows also produce diverse blends of volatile organic compounds (VOCs). These can be emitted from various above- or below-ground plant parts. Here we focus primarily on leaf VOCs that have been predominantly studied in willows and play key roles in their interactions with biotic and abiotic factors (Peacock *et al.*, 2001; Inui *et al.*, 2003; Niinemets, 2010; McCormick *et al.*, 2012; Pearse *et al.*, 2013; Tun *et al.*, 2020; Mezzomo *et al.*, 2023). Willow leaves emit complex VOC blends typically dominated by three main compound classes: green leaf volatiles (GLVs; C6 aldehydes and alcohols), terpenoids (monoterpenes and sesquiterpenes) and aromatics (benzenoids/phenylpropanoids). Depending on species, site conditions and season, emissions may also include substantial quantities of isoprene (Karlsson *et al.*, 2020; Tun *et al.*, 2020). Amino-acid derivatives, such as oximes, have also been reported to occur in willows, although these are typically emitted in minor amounts (Yoneya and Takabayashi, 2013).

Comparative profiling across multiple *Salix* species and hybrids suggests pronounced interspecific and intersex variation in both blend composition and relative compound abundances. Female plants generally emit higher levels of ocimenes and GLVs than males, consistent with the trends observed in non-volatile metabolites and broader evidence of sexual dimorphism in dioecious trees (Randriamanana *et al.*, 2015; Tun *et al.*, 2020). Similar sex-specific differences have been documented in poplars, with environmental stressors further amplifying male–female differences (Maja *et al.*, 2016; Li *et al.*, 2025). VOC profiles also appear to differ between tree- and shrub-form species. In a study done in New Zealand with non-native planted willows, tree-form willows (e.g. *S. alba* and *Salix lasiandra*) produced proportionally more sesquiterpenes than shrubby willows (e.g. *Salix purpurea* and *Salix viminalis*), which, in turn, emitted more monoterpenes and GLVs (Tun *et al.*, 2020). In contrast, a shrubby *Salix cinerea* and a tree-form *S. fragilis* exhibited the inverse pattern in their European range, with the shrub emitting more sesquiterpenes, whereas the tree emitted higher levels of GLVs and monoterpenes (Mezzomo *et al.*, 2024, 2026). The patterns thus seem to vary across studies, highlighting the need for additional research to resolve these relationships and test for them in a phylogenetic context (Maja *et al.*, 2016; Mezzomo *et al.*, 2026).

VOC emissions in willows are strongly shaped by abiotic conditions, particularly temperature and UV radiation. These factors might explain differences in emissions among leaves within the canopy and contribute to seasonal shifts in VOC profiles (McCormick *et al.*, 2019; Karlsson *et al.*, 2020, 2021). For example, emissions of isoprene, ocimene and other VOCs in temperate willows typically peak in summer, probably owing to higher temperatures and increased radiation (Karlsson *et al.*, 2021; Mezzomo *et al.*, 2025). Such temperature- and light-dependent patterns are common in plants, especially for terpenoids, whose biosynthesis is tightly linked to light-driven metabolic processes (Niinemets *et al.*, 2010; Loreto and Fineschi, 2015). Higher temperatures can further enhance emissions by increasing enzymatic activity, facilitating transport of compounds from production sites, promoting volatilization from storage tissues and influencing stomatal conductance (Malik *et al.*, 2023).

High-light conditions also promote the emission of specific monoterpenes, including (*E*)-β-ocimene, α-pinene, β-pinene and β-myrcene, which are frequently detected in willow VOC blends and are associated with mitigation of thermal and oxidative stress (Tollsten and Knudsen, 1992; Lehrman *et al.*, 2013). These responses are particularly pronounced in willows inhabiting open riparian and high-elevation environments, where intense solar radiation and large temperature fluctuations are common (Peacock *et al.*, 2001; Loreto and Fineschi, 2015; Sharkey and Monson, 2017). Elevation shapes willow VOC chemistry further through combined effects of increased UV exposure, lower temperatures, reduced atmospheric pressure and altered biotic interactions (Simin *et al.*, 2022). High-elevation willows often exhibit elevated constitutive emissions of protective VOCs, such as isoprene, which help to buffer oxidative and UV-induced stress (Loreto and Fineschi, 2015; Simin *et al.*, 2022).

Beyond temperature and light conditions, willow VOC emissions are modulated by water and resource availability. Severe drought generally suppresses VOC emissions by constraining photosynthesis, whereas moderate water stress can enhance VOC production in some species, reflecting adaptation to local moisture regimes (Niinemets, 2010). Soil nutrient status, particularly nitrogen and phosphorus availability, also shapes VOC biosynthetic capacity by regulating growth–defence trade-offs, thereby affecting both emission rates and blend composition across natural willow populations (Ormeño and Fernandez, 2012).

VOCs mediate critical within- and between-plant communication (Fig. 4), particularly via key volatiles, such as (*E*)-β-ocimene and GLVs, that mediate systemic priming, allowing undamaged tissues or plants to pre-activate the synthesis of defensive metabolites (Tun *et al.*, 2020). In willows, many of the VOCs induced by herbivory are involved in priming. This mechanism helps the plant to enhance resistance to subsequent attacks and reduce critical tissue loss. Across three field experiments using *Salix exigua* and *Salix lemmonii*, exposure to volatiles from a damaged neighbour reduced herbivore damage by 21–45 % on the receiver plant (Pearse *et al.*, 2013). Bagging of wounded branches eliminated the signalling effect, demonstrating that the cue is airborne and not root-borne or tactile.

**Fig. 4.**
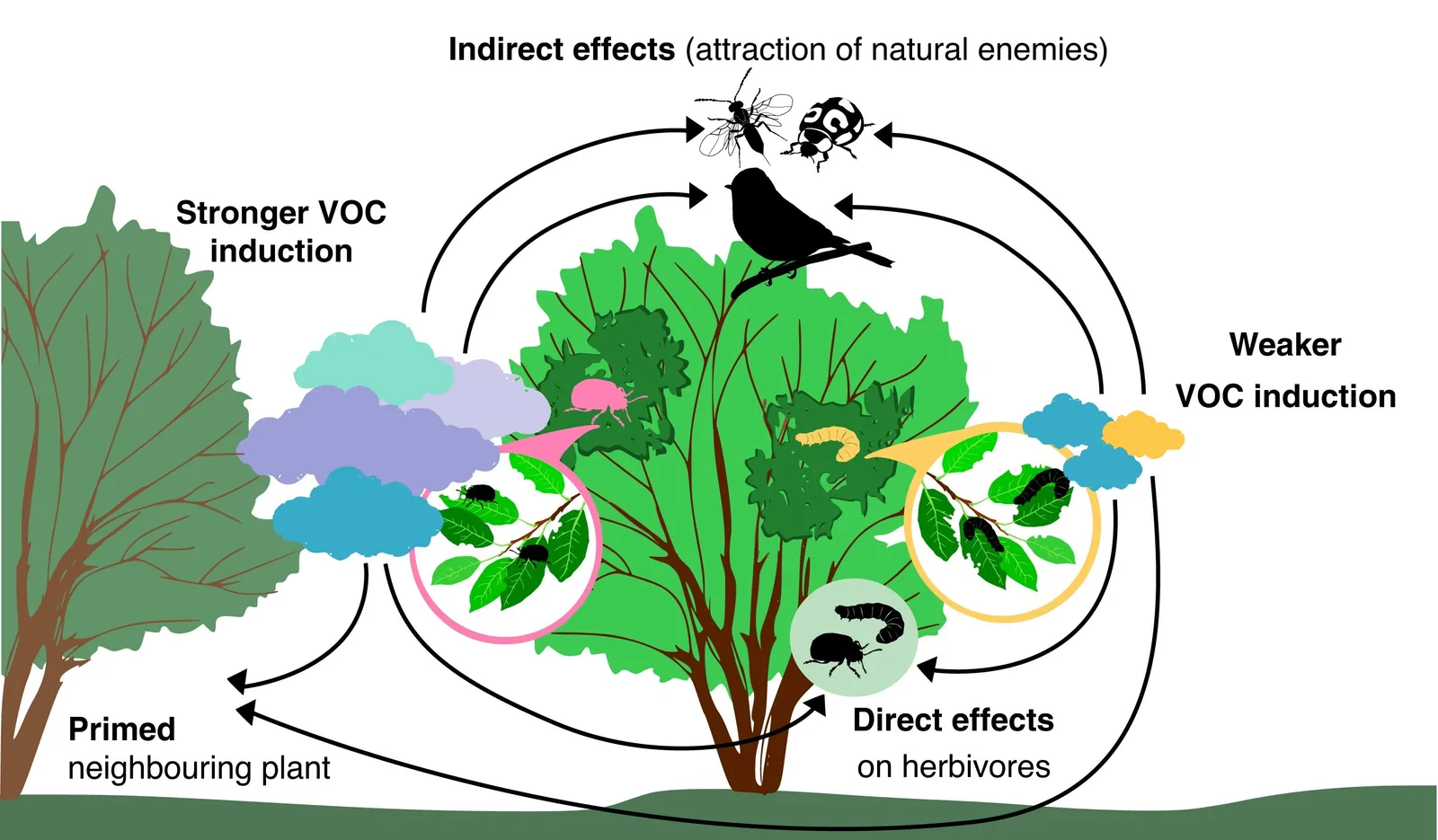
Conceptual overview of volatile organic compounds (VOCs) mediating tri-trophic interactions in willows. Herbivory on a focal willow triggers the emission of VOCs whose composition, diversity and total emission rates vary depending on herbivore identity. VOCs emitted by willows can directly deter herbivores and reduce their performance or attract their natural enemies, including both invertebrates and vertebrates. VOCs may also disperse to neighbouring willows, where they prime defensive responses. The extent to which herbivore-specific VOC blends and their induction strength shape ecological interactions, attract specific predators and, ultimately, influence willow fitness remains to be explored further.

Many willow VOCs also directly influence interactions with insect herbivores. Constitutive volatile emissions in willows are dominated by isoprene, monoterpenes and sesquiterpenes that provide defence against generalist herbivores and some early-season specialists (Gershenzon, 1994; Bekaert *et al.*, 2012). In contrast, other specialists can use constitutive emissions of willow VOCs as feeding or oviposition cues (Austel *et al.*, 2014; Braccini *et al.*, 2015). The net effect on specialist herbivores, whether attractive or repellent, appears to be determined by the relative emission levels of individual VOCs (Ling *et al.*, 2022). The constitutive VOC profile is dynamically supplemented by inducible emissions (Heil, 2014), which can repel generalist herbivores, such as the green peach aphid, because they indicate a defended, low-nutrient host (Ahmed *et al.*, 2019).

Herbivore feeding mode, life stage and intensity of feeding (i.e. herbivory damage) differentially activate biosynthetic pathways, resulting in complex inducible volatile blends largely tailored to the attacking organism (Heil, 2014; McCormick *et al.*, 2014*a*). Mechanical damage alone tends to trigger more generalized VOC responses, underscoring the central role of herbivore-associated molecular pattern recognition and specific plant-herbivore defence induction (Moreira *et al.*, 2012; Mezzomo *et al.*, 2026). Chewing herbivores commonly elicit strong upregulation of monoterpenes, such as (*E*)-β-ocimene and linalool, together with a suite of GLVs that often includes (*E*)-hexenal and (*E*)-3-hexen-1-ol acetate in willows and poplars (Danner *et al.*, 2011; Mezzomo *et al.*, 2023, 2024). Leaf beetle larvae that skeletonize leaves and cause large surface wounds trigger particularly pronounced responses (Lehrman *et al.*, 2013; Mezzomo *et al.*, 2023, 2024).

Conversely, sap-sucking herbivores, such as aphids, mites or whiteflies, typically trigger less pronounced changes in VOC emissions, predominantly mediated via salicylic acid signalling, in contrast to the jasmonic acid pathway that is characteristically activated by chewing herbivores (Danner *et al.*, 2011; Tun *et al.*, 2020; Mezzomo *et al.*, 2023, 2024). Together with possible host manipulation, this might be one reason why sap-sucking herbivores typically elicit limited induced responses in willow VOCs (Niinemets *et al.*, 2010, McCormick *et al.*, 2012; Mezzomo *et al.*, 2023, 2024). Suppression of willow VOCs has been described in response to feeding by giant willow aphids (*Tuberolachnus salignus*) (Tun *et al.*, 2020).

Gall formation, in particular, may generate VOC responses that differ from those induced by free-feeding chewers or sap-sucking herbivores. Gall-inducing sawflies and midges can alter host physiology by co-opting hormonal pathways involved in tissue growth and differentiation. This manipulation can create nutritionally enriched tissues and alter the composition of non-volatile metabolites (Nyman and Julkunen-Tiitto, 2000). However, direct evidence for how gall inducers affect willow VOCs remains scarce. Available evidence suggests that gall-associated herbivory does not suppress VOC emissions uniformly. In the Arctic willow *Salix myrsinites*, eriophyoid mite infestation did not affect isoprene emissions, but stimulated DMNT [(E)-4,8-dimethyl-13,7-nonatriene], sesquiterpene and GLV emissions from both galled and surrounding tissues (Swanson *et al.*, 2021). These findings suggest that gall-inducing arthropods might reshape willow VOC signalling, but the direction and ecological consequences of these changes remain poorly understood. Related work on the willow-feeding sawfly *Nematus oligospilus*, which does not induce galls, further suggests that host manipulation by willow-associated sawflies might begin at oviposition. Egg deposition on *Salix babylonica* activates jasmonic acid signalling and induces a volatile blend dominated by (E)-β-ocimene and (E,E)-α-farnesene, distinct from both feeding-induced and undamaged profiles (Dávila *et al.*, 2023).

Herbivory by different insect species on willows is likely to affect not only the composition of VOCs but also their production over time, as suggested by evidence from poplars (McCormick *et al.*, 2012, 2014*a*). The temporal sequence of induction often involves an early GLV burst followed by slow but long-term terpenoid release. This release pattern forms a mechanistic basis for tri-trophic dynamics, because predators exploit both the fast, damage-linked GLVs and the slower, jasmonic acid-dependent terpenoids as foraging cues, and different predator taxa might specialize on detecting different temporal phases (McCormick *et al.*, 2012).

Herbivore-induced volatiles can attract both invertebrate predators and parasitoids (coccinellids, lacewings, parasitic and predatory wasps, and ants) and vertebrate insectivores (birds) and alter herbivore pressure (McCormick *et al.*, 2012). Critically, this attraction is not generic; predators exploit species- and herbivore-specific volatile fingerprints, and different predator taxa integrate VOC cues differently (Amo *et al.*, 2013; Hermann and Thaler, 2014; Mrazova and Sam, 2018; Mezzomo *et al.*, 2026). For instance, invertebrate predators seem to respond most strongly to highly specific VOC signatures. In contrast, bird predators may integrate volatile cues with visual traits of herbivory, such as changes in leaf reflectance (Mrazova and Sam, 2018; Mezzomo *et al.*, 2026). Pioneering work by Yoneya and Takabayashi (2013) characterized volatile-mediated networks in willows, using a model system including the heartleaf willow tree (*Salix eriocarpa*), a leaf beetle (*Plagiodera versicolora*) and a predatory ladybird (*Aiolocaria hexaspilota*). This work demonstrated high specificity in the use of herbivore-induced VOCs by herbivore natural enemies and recognized the potential role of minor compounds, such as N-containing oximes, in mediating natural enemy attraction. In poplar trees, aldoximes and their resulting nitriles are known to be involved in both direct and indirect defence (Irmisch *et al.*, 2014; McCormick *et al.*, 2014*c*), highlighting the importance of minor compounds in VOC-mediated plant–insect interactions (McCormick *et al.*, 2014*b*).

Plant–herbivore–predator interactions can also involve complex processes, such as honeydew deposition. For instance, aphid attack in willows is linked to prolific honeydew production that attracts honeydew foragers (e.g. bees and flies), ant–aphid mutualists and predators seeking aggregated prey (Tun *et al.*, 2020, 2021). Honeydew VOCs play important roles in attracting these species (Peach *et al.*, 2019; Hung *et al.*, 2020). In the case of bees, willow honeydew VOCs can be traced all the way into honey, acting as a botanical origin biomarker (Jerković *et al.*, 2010). These interactions reveal that VOC signalling operates within a far richer multi-trophic context than simple herbivore–predator systems.

Willow VOCs also shape phyllosphere microbial communities, with antimicrobial monoterpenes reducing pathogen loads and modifying microbe–herbivore interactions (Salih *et al.*, 2024). Such interactions might potentially alter the outcome of herbivore attack by changing microbe-mediated defences or nutrient availability. This dimension of VOC-mediated interactions remains understudied but represents an important frontier for understanding the function of VOCs beyond canonical tri-trophic networks.

Following hybridization, willow VOC profiles generally mirror patterns observed for non-volatile specialized metabolites, tending to be intermediate between those of the parental species (Tun *et al.*, 2020). Nevertheless, VOCs appear to constitute a largely independent axis of chemical variation, showing only weak correlations with other defensive traits (Mezzomo *et al.*, 2023, 2024; Leong *et al.*, 2024). Given their strong upregulation by specialized leaf beetles, VOCs might potentially represent a complementary defence to salicinoids that these leaf beetles can circumvent (Orians *et al.*, 1997; Volf *et al.*, 2015*a*; Mezzomo *et al.*, 2023, 2024). Comparative studies of VOC profiles across multiple willow species in a phylogenetic framework remain scarce, but available evidence indicates a limited phylogenetic signal (Leong *et al.*, 2024; Mezzomo *et al.*, 2024). Rather than exhibiting complete overdispersion with species-specific VOC chemistries, distantly related willows often cluster according to similarities in herbivore-induced VOC responses (Mezzomo *et al.*, 2024). Whether such clustering enhances interspecific plant–plant communication or improves the attraction of natural enemies of shared herbivores requires future research.

## WILLOW CHEMISTRY AND MUTUALISTIC INTERACTIONS

In addition to serving as defences, specialized metabolites also help willows to maintain mutualistic interactions with other organisms. In willows, these interactions primarily involve mycorrhizal fungi, bacteria, and insect pollinators. Colonization of willow roots by ectomycorrhizal and arbuscular mycorrhizal fungi can enhance early growth, root biomass, stress tolerance and nutrient assimilation (Van der Heijden, 2001; Baum *et al.*, 2018). It also induces pronounced shifts in leaf metabolites, as demonstrated for *S. purpurea* by Aliferis *et al.* (2015). Their study documented an upregulation of the porphyrin and chlorophyll biosynthetic pathways, probably contributing to increased leaf carbohydrate levels and decreased sugar alcohols. In addition, they observed upregulation of the biosynthetic pathway producing α-lineolate, the principal precursor of jasmonic acid, which regulates growth, development and stress-induced responses. This might be associated with the concurrent upregulation of flavonoid, phenolic glucoside and terpenoid biosynthesis reported by the authors.

Since this initial study, further research has linked willow–arbuscular mycorrhizal associations to additional chemical and physiological changes, including reduced emissions of sesquiterpenes, increased antioxidant enzyme activity during stress, and other specific responses (Almeida-Rodríguez *et al.*, 2016; Galotta *et al.*, 2025). Arbuscular mycorrhizal fungi-induced responses also occur in roots of *S. viminalis*, where organic acids, such as α-linolenic acid, arachidonic acid and 5-methoxysalicylic acid, significantly increase and promote degradation of harmful organic pollutants in the rhizosphere, benefitting both the plant and the fungi (Li *et al.*, 2023).

Willow root exudates also influence the overall composition of rhizosphere microbial communities (Yergeau *et al.*, 2014) and might affect interactions with bacteria that contribute to the rhizoremediation capacity of willows in toxic or contaminated soils (Pagé *et al.*, 2015). However, the mechanisms by which willow root exudates, such as salicylic acid derivatives, affect rhizosphere bacteria and whether these effects ultimately influence willow performance remain largely underexplored (Yergeau *et al.*, 2014).

Willow floral chemistry mediates mutualistic interactions with pollinators through VOCs emitted from catkins (Dötterl and Gershenzon, 2023). Floral blends are dominated mainly by isoprenoids and benzenoids, with aldehydes, monoterpenes and ketones also contributing to pollinator attraction (Füssel *et al.*, 2007; Burger *et al.*, 2013, 2021). Across the genus, compounds such as trans-β-ocimene and 1,4-dimethoxybenzene explain much of the difference between species, suggesting that a small number of volatiles can contribute disproportionately to interspecific variation in floral scent profiles (Füssel *et al.*, 2007).

Floral VOCs show pronounced sexual dimorphism, although its direction differs among species. In *Salix caprea*, male catkins emit substantially more scent than females and contain higher relative proportions of 1,4-dimethoxybenzene and methyl salicylate (Dötterl *et al.*, 2014). In *S. fragilis*, males emit more trans-β-ocimene and 1,4-dimethoxybenzene, whereas females emit relatively higher amounts of D-limonene and D-verbenone (Füssel *et al.*, 2007). In contrast, in *S. purpurea*, males are richer in terpenoids, whereas females emit higher levels of benzenoids, such as ethyl benzoate (Keefover-Ring *et al.*, 2022). This variation suggests that floral sexual dimorphism in willows might not be governed by a single shared mechanism but could have diverged independently across lineages. In *S. purpurea*, this dimorphism appears to have a polygenic basis, with multiple quantitative trait loci associated with floral chemical traits and several loci co-localizing with sex chromosomes or genes involved in VOC biosynthesis (Keefover-Ring *et al.*, 2022). This pattern is consistent with the idea that floral scent can evolve semi-independently in males and females and could contribute to sex-specific interactions with pollinators.

Emissions of floral VOCs are also temporally dynamic. In *S. caprea*, total VOC emissions peak during the day and increase with temperature, while scent composition shifts between daytime and night-time pollinator activity. Bee-attractive compounds, such as 1,4-dimethoxybenzene, are proportionally more abundant during the day, whereas moth-attractive lilac aldehyde isomers increase at night (Jürgens *et al.*, 2014; Dötterl and Gershenzon, 2023). Finally, floral VOCs are not used exclusively by pollinators. Leaf-galling sawflies can also respond to catkin scent when selecting host plants (Kehl *et al.*, 2010).

## FUTURE PERSPECTIVES

We propose that application of modern bioinformatics and metabolomics approaches to study willow metabolomes can provide new insights into the ecological and evolutionary roles of specialized metabolites in willows and plants in general. Untargeted metabolomics now enables measurement of thousands of metabolites from a single sample (Sedio, 2017; Walker *et al.*, 2022). Using mass spectra, spectral libraries and machine learning, metabolites can be classified into chemical classes and pathways, extending research beyond the compounds traditionally studied in willows and enabling the exploration of processes across a large part of the willow metabolome. The same or related tools can also be used to predict molecular formulas, fingerprints and structures. This allows inference of molecular properties of the measured metabolites correlated with their bioactivity (Walker *et al.*, 2023). These data can be transformed into multidimensional indices of phytochemical diversity, facilitating functional comparisons of chemical strategies among plants across environments (Walker *et al.*, 2022, 2023; Chadwick *et al.*, 2026). This integrative framework offers a way to connect chemical diversity with ecological concepts explaining patterns in plant functional traits (Webb *et al.*, 2002; Cavender-Bares *et al.*, 2004). Through this lens, we can predict how environmental filtering promotes functional similarity or, conversely, how selection drives functional divergence, processes that might help to explain the ecological success and adaptability of willows or other species-rich plant lineages (Volf *et al.*, 2023, 2024).

Likewise, volatilomics, a branch of metabolomics focused on VOCs, is also rapidly growing. Advances in gas chromatography–mass spectrometry and proton-transfer reaction mass spectrometry have enabled high-resolution detection of complex volatile blends (Tholl *et al.*, 2021). Artificial intelligence (AI) and machine-learning approaches, including random forests, support vector machines and deep learning, greatly enhance volatile analysis by enabling automated peak detection, deconvolution of co-eluting compounds, pattern recognition across large sample sets and predictive modelling of VOC emissions in varying ecological or environmental conditions (Betancourt-Arango *et al.*, 2024). These tools help to identify subtle chemical signatures, classify plant traits or stresses based on VOC fingerprints and uncover non-linear relationships often missed by traditional statistical methods (Breiman, 2001; Gamboa *et al.*, 2021). Together, these approaches significantly increase the accuracy, scalability and ecological insight gained from plant VOC data.

Only within the last decade has it become possible to resolve the phylogenetic relationships in *Salix*. Advances in next-generation sequencing now enable the construction of well-supported phylogenies that can be used to interpret patterns in willow chemistry within phylogenetic and evolutionary frameworks (Wagner *et al.*, 2020). Remaining challenges include the reconstruction of evolutionary histories in lineages characterized by high ploidy levels and reticulate evolution (Marinček *et al.*, 2025). Nevertheless, these recent developments provide an unprecedented opportunity to place the chemical ecology of willows within the broader evolutionary context of the entire family (Ogutcen *et al.*, 2024). Notably, willows represent one of the few temperate analogues to species-rich tropical plant genera that have been studied extensively for the evolution of chemical defences (Volf *et al.*, 2019). Given that the basal members of the Salicaceae are tropical species, this opens the door to comparative studies examining how different selection pressures have shaped the metabolomes of tropical versus temperate taxa and whether certain preadaptations found in tropical lineages contributed to the diversification of willows at higher latitudes.

## CONCLUSIONS

The exceptional diversity of *Salix*, its pervasive ecological interactions across various elevations and habitats and its chemically rich yet evolutionarily labile chemistry continue to make willows one of the most informative model systems for studying plant chemical ecology and the evolution of phytochemical diversity. Willows uniquely link broad ecological valence with frequent hybridization, polyploidy and reticulate evolution (Wagner *et al.*, 2023; Marinček *et al.*, 2024), which are key processes shaping the evolution of plants and their metabolomes (Orians, 2000; Gaynor *et al.*, 2020; Wu *et al.*, 2024). At the same time, emerging ‘omics’ approaches are rapidly expanding the portion of the willow metabolome that can be quantified and interpreted in a functional and evolutionary context (Volf *et al.*, 2023; Renoult *et al.*, 2025). By integrating these tools with experiments and comparative field studies, future work can move beyond a small set of canonical compounds to a more holistic view of chemical strategies, revealing how metabolic constraints, environmental filtering and biotic interactions jointly generate and maintain the remarkable chemical variation that underpins willow success (Volf *et al.*, 2024). In this sense, willows remain not only a classic system, but an increasingly powerful one: a species-rich genus through which new methods can illuminate general patterns in chemical diversification, adaptation and evolution in plants.
